# The emerging roles of SUMOylation in pulmonary diseases

**DOI:** 10.1186/s10020-023-00719-1

**Published:** 2023-09-05

**Authors:** Xuyang Zheng, Lingqiao Wang, Zhen Zhang, Huifang Tang

**Affiliations:** 1 Department of pediatrics, The Affiliated Hangzhou First People’s Hospital, Zhejiang University School of Medicine, Hangzhou, 310000 Zhejiang P.R. China; 2 Department of Orthopedics Surgery, The Affiliated Hangzhou First People’s Hospital, Zhejiang University School of Medicine, Hangzhou, 31000 Zhejiang P.R. China; 3 Department of Pharmacology, Zhejiang Respiratory Drugs Research Laboratory, School of Basic Medicial Sciences, Zhejiang University, Hangzhou, 310058 Zhejiang P.R. China

**Keywords:** SUMOylation, Pneumonia, cancer, Hypoxic pulmonary hypertension, Idiopathic pulmonary fibrosis

## Abstract

Small ubiquitin-like modifier mediated modification (SUMOylation) is a critical post-translational modification that has a broad spectrum of biological functions, including genome replication and repair, transcriptional regulation, protein stability, and cell cycle progression. Perturbation or deregulation of a SUMOylation and deSUMOylation status has emerged as a new pathophysiological feature of lung diseases. In this review, we highlighted the link between SUMO pathway and lung diseases, especially the sumoylated substrate such as C/EBPα in bronchopulmonary dysplasia (BDP), PPARγ in pneumonia, TFII-I in asthma, HDAC2 in chronic obstructive pulmonary disease (COPD), KLF15 in hypoxic pulmonary hypertension (HPH), SMAD3 in idiopathic pulmonary fibrosis (IPF), and YTHDF2 in cancer. By exploring the impact of SUMOylation in pulmonary diseases, we intend to shed light on its potential to inspire the development of innovative diagnostic and therapeutic strategies, holding promise for improving patient outcomes and overall respiratory health.

## Introduction

Respiratory diseases are responsible for a significant proportion of global morbidity and mortality, with five of the leading causes of death being chronic obstructive pulmonary disease, acute respiratory tract infections, lung cancer, tuberculosis, and asthma (Schluger and Koppaka 2014). Particularly, the global pandemic of severe acute respiratory syndrome coronavirus 2 (SARS-CoV-2)–induced coronavirus disease, which primarily causes respiratory disease, has been a serious global public health threat since 2019 (Sharma et al. 2021). Meanwhile, chronic respiratory diseases were the third leading cause of death of all deaths in 2017, behind cardiovascular diseases and neoplasms(Collaborators 2020). In order to alleviate the global burden of lung disease, more pathophysiological and molecular mechanisms, as well as novel treatments for each of the major respiratory disorders need to be explored.

Post-Translational Modifications (PTMs) are chemical alterations that occur on protein molecules after their synthesis, involving covalent, enzymatic, or non-enzymatic attachments of specific chemical groups to amino acid side chains. These modifications play a pivotal role in regulating protein function, localization, stability, and activity, thereby significantly expanding the functional diversity of the proteome(Lee et al. 2023). One of the extensively studied PTMs is ubiquitination, a process wherein a small protein called ubiquitin is covalently attached to a target protein. Ubiquitination is orchestrated by a series of enzymatic reactions involving three key enzymes: E1 (ubiquitin-activating enzyme), E2 (ubiquitin-conjugating enzyme), and E3 (ubiquitin ligase)(Song and Luo 2019). A small ubiquitin-like modifier (SUMO) was firstly identified to regulate cell death signals by attaching to the death domain of FAS in 1996(Okura et al. 1996). From then on, more secrets about this protein family are constantly being unearthed. Analogous to ubiquitination, SUMOylation contributes to a diverse array of biological processes. By covalently or non-covalently binding to and detaching from other proteins, SUMOylation can participate in a variety of biological processes, such as genome replication and repair, transcriptional control, protein stability, and cell cycle progression(Chang and Yeh 2020).

Accumulating evidence has established the critical physiological and pathological functions of SUMOylation. Perturbation or deregulation of a SUMOylation and deSUMOylation status has been summarized in numerous diseases, including cancers, cardiovascular diseases, skeletal muscle diseases, neurodegenerative diseases, and diabetes(Bawa-Khalfe and Yeh 2010; Guo et al. 2004; Lhatoo et al. 2015; Mendler et al. 2016; Namuduri et al. 2017). Here, we reviewed multiple studies linking SUMOylation to lung diseases in order to identify novel concepts for the clinical development of therapeutic protocols for lung diseases.

### Introduction of the SUMOylation process

In mammalian cells, five SUMO isoforms have been identified: SUMO1, SUMO2, SUMO3, SUMO4, and SUMO5. SUMO2 and SUMO3 are generally referred to as SUMO2/3 since they are 97% identical and just 46% comparable to SUMO1(Gareau and Lima 2010; Saitoh and Hinchey 2000). SUMO1, SUMO2, and SUMO3 are widely expressed in human tissues, whereas SUMO4 and SUMO5 have restricted expression patterns and have not been thoroughly investigated(Celen and Sahin 2020; Liang et al. 2016; Bohren et al. 2004).

**Fig. 1:**
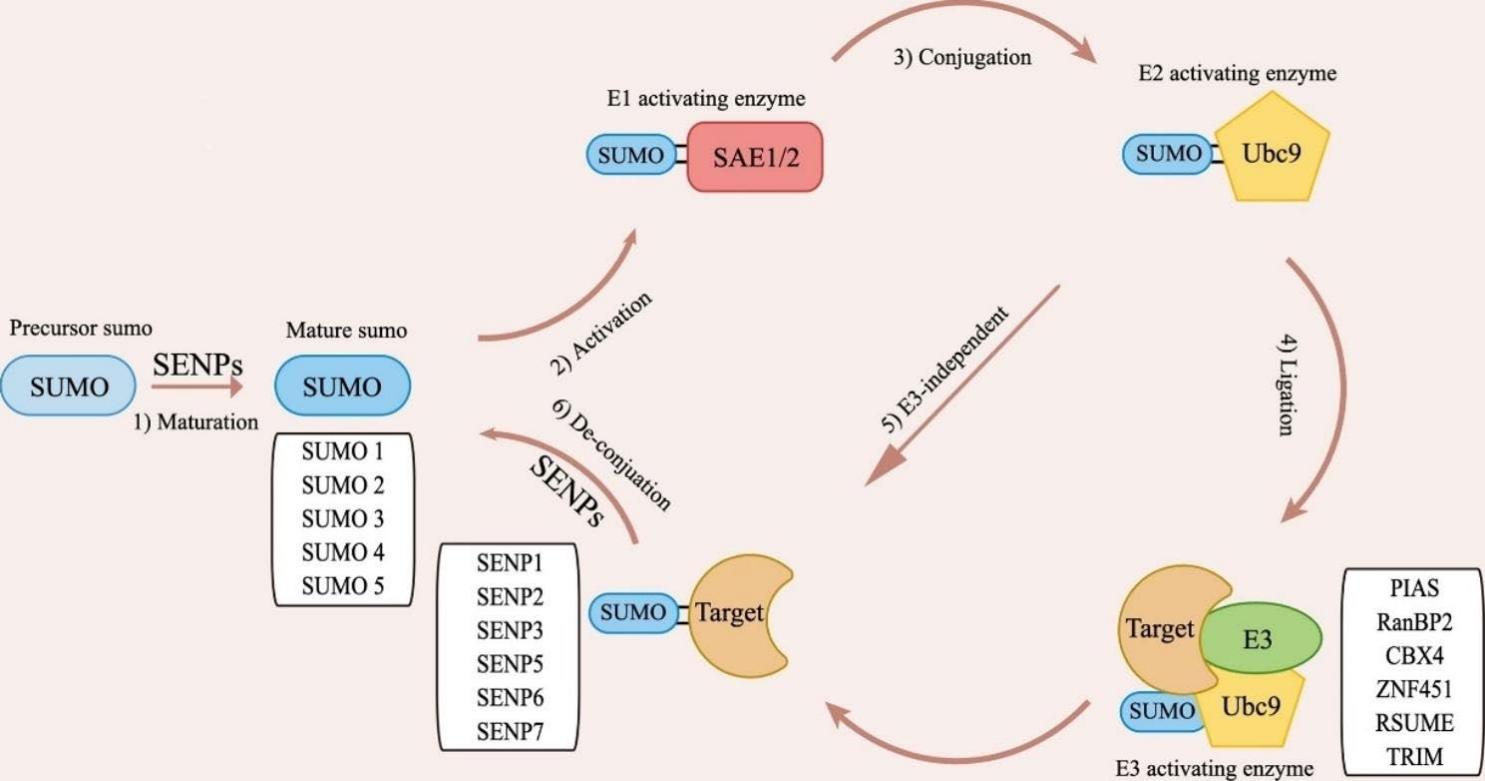
The SUMO pathway 1) The precursor SUMO is converted to mature SUMO through the cleavage of amino acid by SENPs 2) and 3) SUMO E1 activating enzyme (SAE1/2) activates SUMO and transfers it to the E2 conjugation enzyme Ubc9 4) and 5) SUMO was transferred to the target proteins directly by UBC9 or with the assistance of an E3 ligase enzyme (PIAS, RanBP2, CBX4, ZNF451, RSUME and TRIM) 6) SENPs deconjugate SUMO from the target proteins

The human genome encodes six members of the Sentrin/SUMO-specific protease (SENP) family: SENP1, SENP2, SENP3, SENP5, SENP6 and SENP7. The distinct subcellular distributions of these SENP proteins appear to serve as a mechanism for regulating their enzymatic activity towards specific substrates. Specifically, SENP1 and SENP2 display a notable concentration at the nuclear envelope, while within the nucleus, they are observed in nuclear foci that partially overlap with promyelocytic leukemia nuclear bodies (PML-NBs). Remarkably, during mitosis, SENP1 and SENP2 undergo redistribution and localize to the kinetochore, suggesting a dynamic regulation of their subcellular localization. Additionally, it is worth noting that SENP2 demonstrates nucleo-cytoplasmic shuttling activity, enabling it to traverse between these compartments. Conversely, SENP3 and SENP5 predominantly reside in the nucleolus, where they exert their enzymatic activity on proteins involved in ribosome maturation. Interestingly, at the cell cycle G2/M transition, SENP5 undergoes translocation and localizes to the mitochondrial surface. SENP6 and SENP7 primarily reside in the nucleoplasm(Nayak and Müller 2014). Through SENPs, SUMOylation and de-SUMOylation exist in dynamic equilibrium. SENPs may also convert precursor SUMO to mature SUMO, which is then conjugated to target proteins via an enzymatic cascade involving the E1 activating enzyme SAE1/2, the E2 conjugation enzyme Ubc9, and the E3 ligase enzymes(Celen and Sahin 2020). Moreover, Ubc9 alone can bind SUMO to targets in vitro, whereas E3 ligase is frequently required for the specificity and productivity of SUMOylation in vivo (Fig. 1). The main SUMO E3 ligases is the family of activated STAT protein inhibitors (PIAS), which includes PIAS1, PIAS2/PIASx, PIAS3, and PIAS4 /PIASy. Other SUMO E3 ligases such as Ran-binding protein 2 (RanBP2), Chromobox4 (CBX4), Zinc finger protein 451 (ZNF451), RWD-containing SUMO enhancer (RSUME) and some members of the tripartite motif (TRIM) protein family(Flotho and Melchior 2013; Cappadocia and Lima 2018), also have been reported.

### Pulmonary diseases

#### Lung development and bronchopulmonary dysplasia (BPD)

Lung development is a multifaceted and dynamic process that can be histologically categorized into four well-defined stages: the pseudoglandular period, canalicular period, terminal sac period, and the alveolar period(Herriges and Morrisey 2014; Shannon and Hyatt 2004).

The study of free SUMO1 in the lung development of newborn rats at 14 days after birth suggested, the levels of free SUMO1 increased on day 4 as compared to day 1, decreased significantly on day 7 compared to day 4, and then remained stable before day 14. Furthermore, the depletion of SENP1 hindered the proliferation and differentiation status and promoted apoptosis of alveolar type (AT) 2 cells(Wan et al. 2019). While AT2 cells are the precursors of AT1 cells during embryonic development and cell damage(Barkauskas et al. 2013). These findings indicated SUMOylation and deSUMOylation were associated with lung development and differentiation. However, the detailed impact of this process remains largely unknown.

The transcription factor CCAAT enhancer binding protein alpha (C/EBPα), belonging to the family of basic leucine zipper (bZIP) transcription factors, assumes a critical role in diverse physiological processes, including normal tissue development, the regulation of cell proliferation and differentiation, as well as lipid metabolism and biosynthesis(Ramji and Foka 2002; Cassel and Nord 2003). C/EBPs exert regulatory function of lung-enriched gene expression in lung epithelial cell lines, among these genes surfactant proteins (SP) A and D are notable, which play a pivotal role in reducing surface tension within the alveoli, thereby facilitating efficient gas exchange(Berg et al. 2006). Another lung-enriched gene is Clara cell secretory protein (CCSP), which actively participates in airway epithelial repair and serves as a protective agent against oxidative stress(Cassel et al. 2000). Additionally, C/EBPs exert control over the expression of the cytochrome P-450 enzyme CYP2B1, responsible for metabolizing xenobiotics and endogenous compounds in the lung(Luc et al. 1996). Importantly, the expression pattern of these genes during lung development led to the formation of specialized cell types in both proximal and distal lung regions, laying the foundation for proper lung function (Chen et al. 2017).

Bronchopulmonary dysplasia (BPD) is characterized by alveolar simplification, surfactant deficiency, and respiratory distress, a prevalent complication that stands as a significant contributor to both mortality and morbidity rates of preterm neonates(Lignelli et al. 2019; Morty 2018). Hyperoxia can induce excessive reactive oxygen species (ROS) considered as the leading cause of lung injury in BPD(Obst et al. 2022; Buczynski et al. 2013). On the rat model of BPD, levels of SUMO1 and SUMOylated C/EBP protein significant elevated, with a negative correlation between SUMOylated C/EBPα protein and SPs. To further block SUMO1, resulting in increased expression of C/EBP and SPs. Co-immunoprecipitation assays revealed that SUMOylation is critical in regulating the cross-talk between C/EBP and transforming growth factor (TGF) β2, which is a growth factor that inhibits AT2 cell differentiation during lung maturation(Zhu et al. 2020). The findings suggest that SUMOylation of C/EBP also act as a regulatory mechanism in BPD.

Sirtuin1(SIRT1) promoted the deacetylation of p53, thereby reducing oxidative stress-induced injury in peripheral blood mononuclear cells (PBMCs) of newborns and the lung of neonatal rats(Yang and Dong 2021). Oxidative stress may change the post-translational modifications including SUMOylation, and decrease the activity of SIRT1which associated with the development of BPD(Tan et al. 2018). Additionally, ROS level and protein expression of SENP1 increased in PBMCs from BPD infants. In contrast, the expression of SIRT1 decreased in the nucleus and increased in the cytoplasm, followed by a rise in acetylp53 (ACP53) expression. Furthermore, in the hyperoxic alveolar epithelial cell injury model, they discovered that hyperoxia could elicit the same pattern of variation in the SENP1-SIRT1 pathway observed in infants with BPD, and hyperoxia-induced changes could be alleviated by silencing SENP1. Taken together, these findings suggested that SENP1 was critical in hyperoxia-induced lung injury by regulating the expression and nucleoplasmic distribution of SIRT1 through deSUMOylation, which inhibits its deacetylase activity and ultimately promotes cell apoptosis(Dong et al. 2021). As such, Inhibition of SENP1 represents a promising avenue for future therapies of BDP.

#### Lung infections

Except normal lung development and impaired lung development, SUMOylation also is being recognized as a crucial pathway for cell homeostasis and health. When the host is infected with bacteria or viruses, there is a noticeable change in global SUMOylation. Pathogens have developed diverse strategies to counteract the host defense mediated by SUMOylation, as an adaptation(K et al. 2021; Cruz-Herrera et al. 2018). They can directly target enzymes of the SUMOylation pathway or disrupt the SUMOylation dynamics of proteins involved in the immune response against pathogens. Additionally, some pathogens can utilize the host’s SUMOylation machinery to modify their own proteins, thereby aiding in the amplification and sustenance of the infection(Ribet and Cossart 2018).

The respiratory tract is a common site for human adenoviruses (HAdV) infection, which can result in pharyngitis or pneumonia. Promyelocytic leukemia nuclear bodies (PML-NBs) are nuclear dot‐like multi‐protein complexes found in virtually all cells. These complexes resemble nuclear dots and contain multiple cellular proteins such as PML, Sp100, Daxx, and SUMO(Hofmann et al. 2021; Regad and Chelbi-Alix 2001),which contribute to viral replication and host antiviral defense. Adenovirus early region 1 A(E1A) is the first viral gene expressed after infection and known as a viral molecular hub in HAdV(Berk 2005). The interaction between E1A and UBC9 could interfere with polySUMOylation, which refers to the addition of SUMO proteins to a single target protein, but not affect the more widespread SUMO modification or the oncogenic transformation of host cells(Yousef et al. 2010). Intriguingly, viral replication centers (RCs) are discovered juxtaposed to PML‐NBs with antiviral effects in HAdV-infected cells(Doucas et al. 1996). Stubbe et al. (2020). Early region 4 open reading frame 6 (E4orf6) proteins and E1B 55-kDa (E1B55K) are two early products after HAdV infection and have been shown to possess E3 SUMO ligase activity(Fiedler et al. 2022; Muncheberg et al. 2018; Pennella et al. 2010). In addition, the viral protease of HAdV (AVP) has been reported to be similar in structure and function to SENP(Wimmer and Schreiner 2015; Li and Hochstrasser 1999).

COVID-19 is a newly emerging human infectious disease caused by severe acute respiratory syndrome coronavirus 2 (SARS-CoV-2)(Ziegler et al. 2020). SARS-CoV and SARS-CoV-2 belong to the Betacoronavirus genus in the Coronaviridae family and are pleomorphic RNA viruses. The SARS-CoV-2 genome has a sequence homology of 77.5% with SARS-CoV(Kim et al. 2020). It has been reported that the coronavirus N protein, which plays an essential role in replicating genomic RNA and nucleocapsid assembly, has a motif for binding human Ubc9 and could be modified by SUMO1 at the lysine 62 residue, resulting in the homo-oligomerization of the N protein. SUMOylation of this protein could play a functional role in SARS-CoV replication as that homo-oligomerization is essential for the proper activity of the N protein(Fan et al. 2006; Li et al. 2005). Therefore, disrupting SUMOylation of the N protein could potentially hinder its homo-oligomerization, subsequently impacting its normal functionality and inhibiting coronavirus replication. Given the substantial sequence similarity observed in the N protein across various coronaviruses, it is reasonable to postulate that the N protein of SARS-CoV-2 might undergo SUMO modification, and potentially possess similar or identical SUMOylation sites as those identified in other coronaviruses(Zhou et al. 2020a; Marra et al. 2003). Further experimental studies and investigations are imperative to ascertain the actual occurrence of SUMOylation on the N protein in SARS-CoV-2. Such research endeavors will provide critical insights into the molecular mechanisms underlying viral biology and potentially targeted therapeutic interventions against COVID-19.

In coronavirus-infected cells, angiotensin-converting enzyme 2 (ACE2) is the cellular receptor protein for SARS-CoV-2(Ziegler et al. 2020; Shang et al. 2020). Recently, it has been reported that ACE2 can interact with SUMO3 at lysine (K) 187. This interaction impedes the K48-linked ubiquitination of ACE2, thereby suppressing its subsequent degradation as a cargo receptor that is dependent on Toll-interacting protein (TOLLIP)-mediated autophagy. Additionally, the E3 SUMO ligase PIAS4 promotes the SUMOylation and stabilization of ACE2, while the deSUMOylation enzyme SENP3 reverses this process. TOLLIP deficiency results in ACE2 stabilization and host susceptibility to SARS-CoV-2(Jin et al. 2022). Their results highlight a potential strategy for preventing SARS-CoV-2 infection: inhibition of ACE2 SUMOylation promote its ubiquitination, and thereby augment the selective autophagic degradation of ACE2.

Bacterial pneumonia is a global health burden and Gram-negative bacteria such as *Klebsiella* pneumoniae infections are a major contributor. Unwarranted inflammation is suggested as an essential cause of mortality(Mizgerd 2008). Interleukin (IL)-10 from lung myeloid-derived suppressor cells (MDSCs) directly suppresses proinflammatory cytokine production to prevent persistent lung inflammation. Previous reports have been shown that elevated levels of mitochondrial-DAMP cardiolipin (CL) block IL-10 production from lung MDSCs through the CL-mediated K107 SUMOylation of peroxisome proliferator-activated receptor γ (PPARγ)(Pascual et al. 2005; Chakraborty et al. 2017). Garg et al furtherly identified PIAS2 as the E3-SUMOligase responsible for this SUMOylation Garg et al. 2021). Meanwhile, JNK-MAPK mediates CL-mediated PPAR S112 phosphorylation and is necessary for PIAS2 recruitment. A commercially tested peptide inhibitor targeting JNK-MAPK, could block these PTMs of PPARγ, restore IL-10 expression, and improve the survival of murine pneumonia models(Garg et al. 2021). Additionally, it was uncovered that *Klebsiella* pneumoniae infection induces a significant decrease in overall SUMOylation of host proteins in epithelial cells and macrophages to subvert cell innate immunity(Sa-Pessoa et al. 2020). In lung epithelial cells, the detailed analysis further indicated that *Klebsiella* increases the SENP2 in the cytosol by affecting its K48 ubiquitylation and subsequent degradation by the ubiquitin-proteasome. The expression of COP9 signalosome subunit 5 (CSN5) was induced by *Klebsiella* to prevent the NEDDylation of the Cullin-1 subunit of the ubiquitin ligase complex E3-SCF-βTrCP and thus suppressed SENP2 degradation. In macrophages, Toll-like receptor 4 (TLR4)-TRAM-TRIF-induced type I interferon (IFN) signaling via IFN receptor 1 (IFNAR1) controls the decrease in SUMOylation levels triggered by *Klebsiella*, through the action of let-7 microRNAs (miRNAs)(Sa-Pessoa et al. 2020). Their results highlight the essential role of *Klebsiella* polysaccharides in reducing SUMO-conjugated protein levels in epithelial cells and macrophages, which limits the activation of inflammatory responses and promotes the intracellular survival of bacteria in macrophages.

SUMOylation have also been reported in other species of pathogens, including fungi and parasites. In the genome of Aspergillus flavus, a single homologue of the SUMO gene has been identified and designated as AfsumO, which confirms the presence of SUMOylation in this pathogenic filamentous fungus(Nie et al. 2016). This finding is significant in understanding the role of SUMOylation in the biology and pathogenesis of A. flavus. Furthermore, it has been observed that SUMO-dependent mechanisms play a critical role in controlling protein activity, localization, and stability in both lung schistosomula and adult worm stages(Pereira et al. 2011, 2012, 2014). Those discoveries of SUMO-dependent pathways in different pathogens opens up additional possibilities for research into infectious disease pathogenesis.

#### Asthma

Asthma is a persistent inflammatory disease caused by complex gene-environment interactions. Despite extensive research, our understanding of the precise mechanisms by which these interactions ultimately lead to the development of asthma remains limited(Mims 2015). SUMO1 and SUMO2/3 expression are upregulated in house dust mite (HDM)-induced allergic airway epithelium. When SUMOylation is inhibited, airway inflammation, mucus overproduction, and airway hyperreactivity are all reduced(Liang et al. 2022). Long-form thymic stromal lymphopoietin (lfTSLP) is overexpressed in HDM-induced epithelium (Harada et al. 2009). An increased expression of IfTSLP in the airways is a character of asthma, which has been reported to correlate with an increased expression of type 2 chemokines and disease severity(Matera et al. 2020). The RNA-binding protein muscle excess (MEX)-3B upregulates the expression of lfTSLP by interacting with the lfTSLP mRNA and facilitating its translation. Additionally, chromobox4 (CBX4), an SUMOylation E3 ligase, enhances the transcription of MEX-3B by increasing the SUMOylation of transcription factor II-I (TFII-I)(Liang et al. 2022). These findings implicated the CBX4-TFII-I-MEX-3B-lfTSLP pathway mediated allergic airway inflammation, thereby suggesting CBX4 inhibition as a potential therapeutic protocol for asthma management.

#### Chronic obstructive pulmonary disease (COPD)

Chronic obstructive pulmonary disease (COPD) is an inflammatory lung syndrome characterized by persistent airway obstruction, ultimately leading to compromised airflow and decreased lung functionality (Jones et al. 2017). Cigarette smoke extract (CSE) can induce the generation of excessive oxidative stress from inflammatory cells, which plays critical pathogenic roles in COPD, and tobacco use is the first causal and single most important risk factor for developing the disease(Lopez-Campos et al. 2016).

Significantly increased SUMO1 and Ubc9 expression have been observed in human bronchial epithelial cells (HBEs) exposed to CSE(Zhou et al. [Bibr CR57][Bibr CR76]). Reduced histone deacetylase 2 (HDAC2) activity and expression in COPD peripheral lung and alveolar macrophage leads to inflammatory response enhancement (Barnes 2009). S-Carboxymethylcysteine(S-CMC) is a mucoactive drug used in the treatment of COPD. Song et al. reported that S-CMC reversed the CSE induced down-regulation of HDAC2 expression/activity in a thiol/glutathione (GSH)-dependent manner and increased the sensitivity of steroid therapy(Song et al. 2015). A subsequent study discovered that under physiological conditions, SUMO1 and SUMO2/3 modification happen in HBE cells, and CSE induced SUMO1 modification of HDAC2 in a dose and time-dependent manner. And S-CMC suppressed the expression of IL-8 and upregulated HDAC activity by blocking CSE-induced SUMO1 modification of HDAC2 in the presence of thiol/GSH (Song et al. 2019). These findings offer novel insights that inhibition of HDAC SUMOylation will enhance HDAC activity and thereby suppress COPD-related inflammatory responses.

Members of the cytochrome P450 superfamily, such as Cytochrome P450 1A1 (CYP1A1), have been implicated in induction of oxidative stress, which may be a major risk factor in the etiology of COPD(Antus and Kardos 2015). Increased ROS production and oxidative stress may result from promotion of SUMOylation of CYP1A1 by CSE(Zhou et al. [Bibr CR57][Bibr CR76]).

In alveolar macrophages, the gene expression is known to be altered by inhaled tobacco smoke, one important pathogenesis of COPD(Shaykhiev et al. 2009; Lugg et al. 2022). While microRNAs (miRNAs) play a crucial role in regulating gene expression. The stem-loop structure of pre-miRNAs is cleaved by double-stranded RNA-specific endoribonuclease dicer (DICER), a cytosolic RNA endonuclease, releasing the miRNAs in their mature 20–22 nucleotide single-stranded form(Park et al. 2011). In smokers’ macrophages, DICER was modified by SUMO2/3, and this modification could be prevented by inhibiting Ubc9. SUMOylated DICER has less transcriptional activity may be one reason why smokers’ alveolar macrophages fail to mature appropriately(Gross et al. 2014). So, smoke or CSE promoted SUMOylation maybe the future target to explore the treatment for COPD.

#### Hypoxic pulmonary hypertension (HPH)

Hypoxic pulmonary hypertension (HPH), associated with several diseases, including COPD, interstitial lung disease, and other chronic pulmonary disease. Pathophysiological character of HPH were sustained active vasoconstriction and pulmonary vascular remodeling. The latter involved in thickening, enhanced muscularity, and migration and proliferation of pulmonary artery smooth muscle cells (PASMCs)(Wright et al. 2005; Jin et al. 2014).

Autophagy activation and a phenotypic switch to a synthetic phenotype of PASMCs were found accompanied by significantly increased expression of SUMO1 in HPH mouse models(Jiang et al. 2015). Moreover, the overexpression of SUMO1 under hypoxia has been shown to increase proliferation, migration, and dedifferentiation of PASMCs. The mechanism underlying these effects is believed to be through the regulation of autophagy activation by SUMO1, which induces the SUMOylation of vacuolar protein sorting 34 (Vps34) and the formation of autophagy initiation complex comprised of Beclin-1, Vps34 and autophagy-related gene 14-like protein (Atg14L)(Yao et al. 2019; Lu et al. 2016).

SENP-induced deSUMOylation of hypoxia-inducible factor-1α (HIF-1) has been reported to play a critical role in the significant functional and phenotypic changes that occur during pulmonary hypertension（Zhou et al. 2016, 2008). However, the impact of SUMOylation on HIF-1α stability remains controversial. For example, Bae et al. proposed that SUMO-1 can promote both the protein abundance and transcriptional activity of HIF-1α（Bae et al.^2004^). Similarly, Carbia et al. demonstrated that RSUME may stabilize HIF-1α by increasing its SUMOylation(Carbia-Nagashima et al. 2007). In contrast, other authors have shown that SUMO modification of HIF-1α suppresses its transcriptional activity（Cheng et al.^2007^).

A declined endothelial nitric oxide synthase (eNOS) activity and an increased ROS were the characters of the endothelial dysfunction in the crucial early step in the development of HPH(Murata et al. 2002). Researches indicate that the enzyme arginase 2 (Arg2) plays a critical role in controlling both eNOS production and endothelial function (Hara et al. 2020; G et al. 2020). In human pulmonary microvascular endothelial cells (HPMEC), hypoxia leads to upregulation of Arg2 transcription, diminished eNOS function and vascular dysfunction. while Pandey D et al. suggested Krupple-like factor 15 (KLF15) mediated pulmonary endothelial homeostasis by repressing endothelial Arg2 expression, and the mechanism involves hypoxia-triggered deSUMOylation of KLF15 by SENP1, then KLF15 transfers from the nucleus to the cytoplasm, leading to its ubiquitination-dependent proteasomal degradation(Pandey et al. 2018). Based on these results, KLF15 overexpression or SENP1 blockade could be considered as novel therapeutic strategies for pulmonary hypertension.

#### Idiopathic pulmonary fibrosis (IPF)

Idiopathic pulmonary fibrosis (IPF) is a progressive and fatal interstitial lung disease that is commonly attributed to repeated local micro-injuries to the aging alveolar epithelium. These micro-injuries result in the activation of matrix-producing myofibroblasts, excessive extracellular matrix accumulation, and remodeling of the lung interstitium(Richeldi et al. 2017).

Previous studies have shown that SUMOylation and deSUMOylation play a role in the development of fibrosis in different organs, including the heart, liver, and kidney(Liu et al. 2017; Zhao et al. 2021; Wang et al. 2018; Sun et al. 2022). A recent study revealed that significantly higher expression of SUMO1, SUMO2, and UBC9 in total lung tissue of IPF patients compared to healthy individuals, accompanied by downregulation of SENP1(Yu et al. 2022). TGFβ1 has been implicated in epithelial-mesenchymal transition (EMT) and the development of IPF through various signaling pathways, including the TGFβ1/Mothers against decapentaplegic homolog (SMAD) pathway(Inui et al. 2021; Ghatak et al. 2017). SUMOylated SMAD4 contributes to TGF-β1-mediated EMT, a process implicated in the development of IPF. SUMO-1 inhibitor GA may reduce SUMOylation activity by upregulating SENP1 and thereby prevent the development of IPF(Yu et al. 2022). It is worth noting that PIAS4 is known to suppress TGF-β signaling by increasing the SUMOylation of SMAD3, which is an essential downstream effector of TGF-β signaling(Imoto et al. 2008). Lear et al. identified fibrosis-inducing E3 ligase 1 (FIEL1) up-regulated in the lung tissues of patients IPF, which associated with the down-regulation of PIAS4. The authors further observed that FIEL1 selectively targets and degrades PIAS4, which in turn activates fibrotic signaling in epithelial cells and fibroblasts. Moreover, the authors developed a FIEL1 inhibitor, BC-1480, which was shown to interfere with the FIEL1-PIAS4 pathway and raise PIAS4 levels in murine models. This inhibitor has the potential to reduce fibrosis in IPF patients by restoring the inhibitory effects of PIAS4 on TGF-β signaling(Lear et al. 2016; Lear and Chen 2016).

It has been determined that Lung-resident mesenchymal stem cells (LR-MSCs) may be a source of myofibroblasts, accelerating the development of IPF(Inui et al. 2021; Yang et al. 2021a). Overexpression of SENP1 in LR-MSCs can stimulate the Wingless/Integrated (WNT)/β-catenin and Hedgehog/Glioma-associated oncogene homolog (GLI) signaling pathways by deSUMOylation of critical proteins, and encourage the transformation of LR-MSCs into myofibroblasts, exacerbating the development of pulmonary fibrosis(Sun et al. 2022). As a result, SENP1 has emerged as a promising therapeutic target for the restoration of LR-MSC physiological functions and the treatment of pulmonary fibrosis.

#### Lung cancer

The SUMO pathway is also involved for the formation and progression of cancer, promoting cell proliferation, apoptosis resistance, and the ability for metastasis through controlling proteins implicated in carcinogenesis.

Growing studies suggested SUMO1-3, related lncRNAs and proteins may be the potential prognostic marker for lung adenocarcinoma. SUMO1 overexpression in Non-small cell lung cancer (NSCLC) is linked to a more aggressive tumor phenotype and poorer prognosis, likely through its promotion of NF-κB activity and subsequent regulation of genes involved in tumor cell proliferation, invasion, and metastasis(Ke et al. 2019). Long non-coding RNA (lncRNA) small ubiquitin-like modifier 1 pseudogene 3 (SUMO1P3) has been observed to enhance the migration and invasion of NSCLC cells by downregulating miRNA miR-136(Zhang et al. 2019), which has been implicated as an oncogenic lncRNA in several human malignancies. Its expression has been found to be an independent predictor in patients with lung adenocarcinoma, when compared to SUMO1(Su et al. 2019). Among the lncRNAs, small nucleolar RNA host gene 3 (SNHG3) exerts a positive regulatory effect on the expression of SUMO2 by acting as a molecular sponge for miR-515-5p, has been found to be overexpressed in lung cancer tissues and cells, and is known to promote cell proliferation, migration, invasion, and EMT process (Li et al. 2021).

M2 isoform of pyruvate kinase (PKM2) plays a crucial role in regulating the form of adenosine triphosphate (ATP), elevated levels of PKM2 are frequently observed in lung adenocarcinoma patients (Zahra et al. 2020). It has been indicated that SUMO1 may modify PKM2 to enhance its activity, leading to increased glucose uptake and metabolism. This modification may also alter the function of PKM2, promoting its translocation to the nucleus, where it can modulate gene expression and contribute to tumor growth and survival(An et al. 2018).

YTH domain family of proteins2(YTHDF2) specifically recognizes and binds to the m6A motif within the consensus RR(m6A) CH sequence(Zhu et al. 2014). TCGA dataset analysis suggested that increased expression of YTHDF2 combinate with increased expression of SUMO1, which associated with poor prognosis in patients with lung adenocarcinoma(Hou et al. 2021). In NSCLC cells H1299, YTHDF2 promotes various oncogenic processes such as proliferation, migration, colony formation, and tumor growth. This effect is mediated by SUMOylation of YTHDF2, which enhances its binding affinity to m6A-modified RNAs and subsequently leads to the degradation of specific RNAs(Hou et al. 2021).

In small cell lung cancer (SCLC), E1 activating enzyme SAE1/2, the E2 conjugation enzyme Ubc9, and the E3 ligase enzymes were also overexpressed. SAE2 appears to play an essential role in SCLC tumorigenesis, metastasis, and chemosensitivity, indicating that it could serve as a promising biomarker and therapeutic target for SCLC patients, especially those with high c-Myc expression. SAE2 knockdown reduces tumorigenesis and increases treatment sensitivity. The expression levels of SAE2 may aid in the identification of cancerous tissues, and may be targeted therapeutically to impede cancer progression(Liu et al. 2015). Ubc9 overexpression has been linked to increased migration and invasion in these cells(McDoniels-Silvers et al. 2002; Li et al. 2013). miR-30e can negatively regulate UBC9 expression by suppressing its translation. Interestingly, miR-30e is downregulated in some tumors, suggesting that dysregulation of this miRNA may contribute to the overexpression of Ubc9 in lung cancer(Wu et al. 2009). However, Ubc9 expression may play a different role in primary versus metastatic cancer. Tissue sequence analysis has revealed that Ubc9 expression is increased in primary colon and prostate cancers compared with their normal tissue counterparts, but reduced in metastatic breast, prostate, and lung malignancies compared with their corresponding normal and primary adenocarcinoma tissues(Moschos et al. 2010). E3 ligase enzymes such as PIAS1, PIAS4, also upregulated in NSCLC, degradation or accumulation of the target substrate leads the progression and metastasis of tumors. The inactivation of the promyelocytic tumor suppressor PML leads to cancer susceptibility, while PIAS1 activates the SUMOylation process of PML, leading to its degradation (Wang et al. 1998). Another target focal adhesion kinase (FAK), is a non-receptor tyrosine kinase. The accumulation of FAK in the nucleus has been linked to the progression and metastasis of tumors(Sulzmaier et al. 2014). Constanzo et al. demonstrated that a subset NSCLCs exhibits co-amplification of FAK and PIAS1, and these proteins are enriched in metastatic NSCLCs. The mechanism underlying this phenomenon involves the stimulation of proteolytic cleavage of the FAK C-terminus, focal adhesion maturation, and FAK nuclear localization through the ectopic expression of PIAS1. This process enhances extracellular matrix (ECM) interaction and DNA repair regulation, thereby promoting NSCLC survival and progression(Constanzo et al. 2016).The Slug-E-cadherin axis has emerged as a critical regulatory pathway in NSCLCs, whereby the anomalous overexpression of Slug facilitates the metastasis of cancer. More specifically, the SUMOylation of Slug mediated by Ubc9/PIAS4 recruits HDAC1 and promotes hypoxia-induced NSCLC progression(Hung et al. 2019). SIRT1 is a well-known regulator of cellular processes such as metabolism, aging, and tumorigenesis. Previous studies have shown that SIRT1 is dysregulated in various types of cancer, including lung cancer, and can affect cancer cell proliferation, survival, and migration. SIRT1 acts as a suppressor of cancer cell migration by impeding the EMT process both in vitro and in vivo. Furthermore, disruption of SUMOylation by targeting either Ubc9 or PIAS4 restored SIRT1 expression and favored an epithelial-like phenotype of cancer cells, thus preventing metastasis(Sun et al. 2013). But PIAS3 reduced in squamous cell carcinoma (SCC) patients, which is associated with poor survival rates(Abbas et al. 2015; Kluge et al. 2011). Overexpression of PIAS3 inhibits cell growth and restores the drug sensitivity of human lung cancer cells, which is attributed to the suppression of antiapoptotic molecule Akt phosphorylation(Ogata et al. 2006). The SUMOylation of N-Myc downstream-regulated gene 2 (NDRG2) mediated by ubiquitin ligase RNF4 inhibits the growth, metastasis, and invasion of human lung adenocarcinoma cells(Tantai et al. 2016).

Interestingly, SENPs medicated de-SUMOylation process also overexpressed in patients with NSCLC or SCC. SENP1 has been reported to have a negative correlation with treatment response and could potentially predict chemosensitivity(Mu et al. 2014; Liu et al. 2018; Yang et al. [Bibr CR110][Bibr CR135]). Inhibiting SENP1 can effectively suppress the growth of lung cancer cells by activating A20-mediated ferroptosis(Gao et al. 2022). Another study found that inhibiting SENP1 significantly increases the radiosensitivity of lung carcinoma by promoting ionizing radiation-induced cell cycle arrest, γ-Histone H2A family member X (γ-H2AX) expression, and apoptosis(Wang et al. 2013a). Therefore, SENP1 could potentially serve as an indicator for tumor characteristics and prognosis in NSCLC, which may further improve patient management. Furthermore, the amplification of chromosome 3q26-29 has been recognized as a crucial genomic alteration region in SCC. Within this region, SENP2 has been identified as one of the driver genes and proposed to contribute to the development of lung cancer(Wang et al. [Bibr CR137][Bibr CR138]).

Taken together, these findings suggest that the dysregulation of SUMO process may play a crucial role in the pathogenesis of lung cancers, could potentially serve as viable therapeutic targets in the context of lung cancer.

**Fig. 2:**
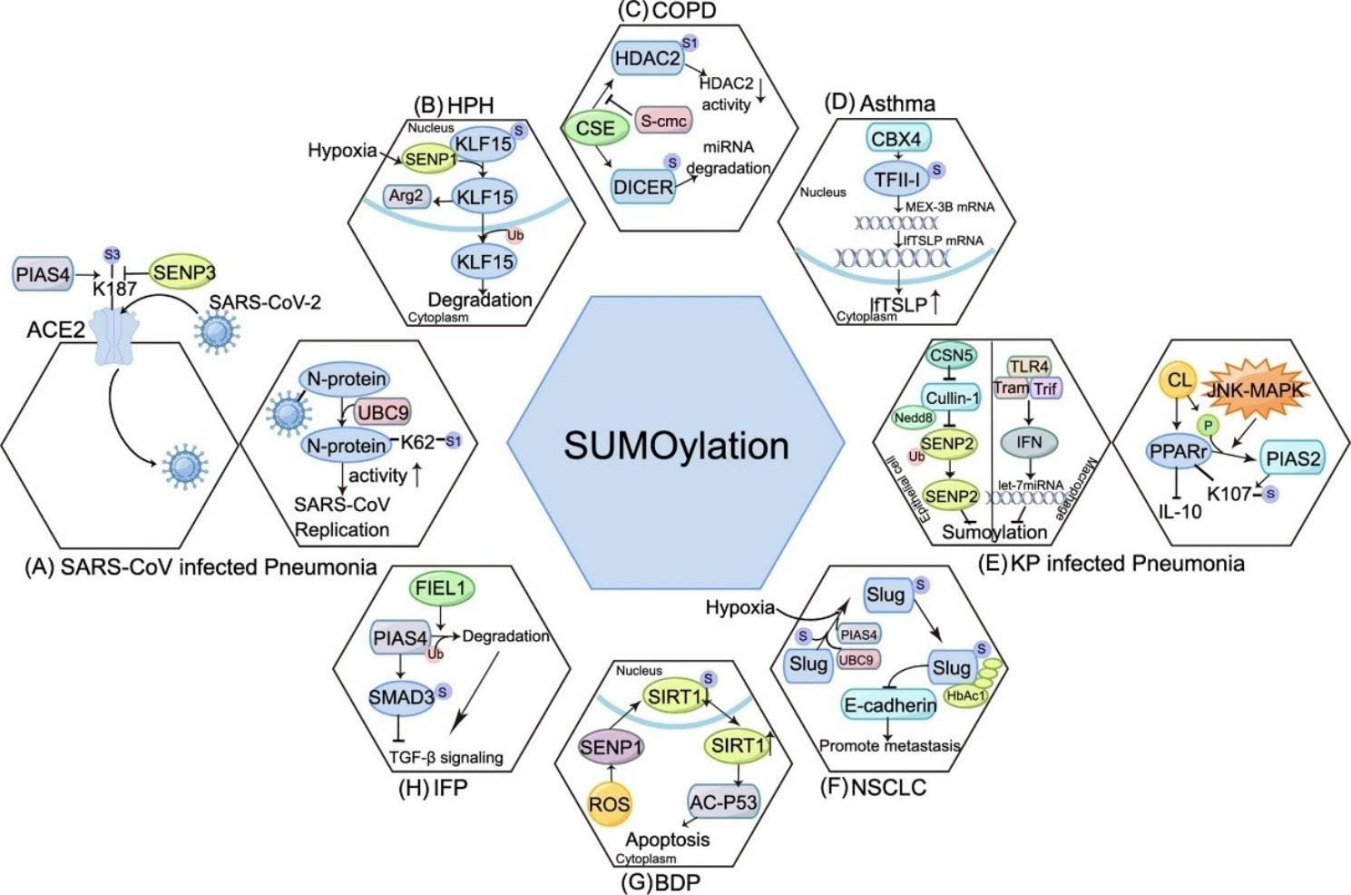
SUMOylation-mediated regulation in pulmonary diseases (A) In SARS-CoV-2 infected cells, the coronavirus N protein could be modified by SUMO1, promoting SARS-CoV replication. In addition, ACE2, the cellular receptor protein for the virus, could interact with SUMO3 by PIAS4 to suppress its degradation; (B) In HPMECs of HPH, SENP1 regulates the abundance and nuclear-cytoplasmic distribution of KLF15 through SUMOylation/deSUMOylation, which is a process sensitive to hypoxia; (C) In HBE cells of COPD, S-CMC can upregulate HDAC activity by blocking CSE-induced SUMO1 modification of HDAC2, making it a potential therapeutic drug for COPD. In smokers’ alveolar macrophages, SUMO2/3 modified DICER inhibits the maturation of miRNAs; (D) In HDM-induced allergic asthma airway epithelium, CBX4 boosts the transcription of MEX-3B by increasing the SUMOylation of general transcription factor TFII-I, which in turn enhances the translation of lfTSLP by binding to its mRNA; (E) In lung MDSCs isolated from mouse models of K. pneumoniae (KP) pneumonia, the CL-mediated K107 SUMOylation of PPARγ inhibits the production of IL-10 through PIAS2 recruitment which requires concurrent activation of PPAR S112 phosphorylation by JNK-MAPK. Additionally, in lung epithelial cells, Klebsiella induces the expression of CSN5 to prevent the NEDDylation of the Cullin-1 subunit of the ubiquitin ligase complex and thus suppresses SENP2 ubiquitylation and subsequent degradation. In macrophages, TLR4-TRAM-TRIF-induced IFN decreases the SUMOylation levels via *let-7* miRNAs; (F) In NSCLCs, Ubc9/PIAS4-mediated Slug SUMOylation and subsequent HDAC1 recruitment lead to the hypoxia-induced lung cancer metastasis; (G) In BDP, SENP1 controls the expression and distribution of SIRT1 by deSUMOylating it in the hyperoxic alveolar epithelial cell damage model, leading to increased Ac-p53 expression levels and promoting cell apoptosis; (H) In lung epithelial cells of IPF, FIEL1 targets PIAS4 and causes its degradation, promoting the SUMOylation of SMAD3 and TGF-β signaling;

## Conclusion

Despite extensive discourse over the past decades on the potential role of the SUMO system in neurodegenerative diseases, cancer, skeletal muscle diseases, and cardiovascular diseases, the limited evidence linked SUMOylation to respiratory diseases. The detailed mechanisms of SUMO proteins in lung disorders and their clinical significance have been summarized here (Fig. 2). Targeting SUMOylation as a therapeutic strategy for lung diseases is still in its early stages, but several studies have shown that inhibition of SUMOylation has the potential to reduce inflammation and oxidative stress in COPD and to induce cell cycle arrest and apoptosis in lung cancer(Song et al. 2019; Bellail et al. 2021). Unraveling the complexities of SUMOylation will help to understand the pathogenesis of lung diseases and design better drugs, ultimately improving the lives of patients suffering from lung diseases.

## Educational aims

The reader will come to appreciate that:

1. Potential role of SUMOylation in lung diseases has provided valuable insights into the pathogenesis and progression of various pulmonary disorders.
2. The pharmacological or other interventions that alter protein SUMOylation can provide new insight into potential treatments for these diseases.

## Directions for future research

The key challenge of SUMOylation inhibitors development for clinical purposes is inhibitor specificity. Long-term inhibition of SUMOylation may have unknown side effects, requiring further safety and efficacy studies. Despite challenges, targeting SUMOylation in diseases offers promising benefits. For example, TAK-981, is a small molecule inhibitor of the SUMO E1 activating enzyme that has shown promising results in preclinical models of acute myeloid leukemia and multiple myeloma(Langston et al. 2021; Kim et al. 2023). As a novel inhibitor of global SUMOylation, topotecan has demonstrated remarkable anticancer, anti-inflammatory, and neuroprotective effects(Bernstock et al. 2017; Mo et al. 2002). Additionally, some research indicated that topotecan may induce SENP1 reduction, producing antileukemic effects(Niu et al. 2022). Besides, SUMO E1 activator drug N106 also serve as a potential therapeutic strategy for treatment of heart failure by increasing SUMOylation of SERCA2a(Kho et al. 2015). So, identifying the perturbation or deregulation of a SUMOylation and deSUMOylation status in disease processes emphasize the need for further investigation on the potential molecular and cellular mechanisms.

## Data Availability

Not applicable.

## References

[CR129] Abbas R (2015). PIAS3 expression in squamous cell lung cancer is low and predicts overall survival. Cancer Med.

[CR116] An S (2018). Small ubiquitin-like modifier 1 modification of pyruvate kinase M2 promotes aerobic glycolysis and cell proliferation in A549 human lung cancer cells. Onco Targets Ther.

[CR80] Antus B, Kardos Z (2015). Oxidative stress in COPD: molecular background and clinical monitoring. Curr Med Chem.

[CR92] Bae SH (2004). Sumoylation increases HIF-1alpha stability and its transcriptional activity. Biochem Biophys Res Commun.

[CR24] Barkauskas CE (2013). Type 2 alveolar cells are stem cells in adult lung. J Clin Invest.

[CR77] Barnes PJ (2009). Role of HDAC2 in the pathophysiology of COPD. Annu Rev Physiol.

[CR8] Bawa-Khalfe T, Yeh ET (2010). SUMO losing balance: SUMO proteases disrupt SUMO homeostasis to facilitate Cancer Development and Progression. Genes Cancer.

[CR139] Bellail AC (2021). Ubiquitination and degradation of SUMO1 by small-molecule degraders extends survival of mice with patient-derived tumors. Sci Transl Med.

[CR27] Berg T, Didon L, Nord M (2006). Ectopic expression of C/EBPalpha in the lung epithelium disrupts late lung development. Am J Physiol Lung Cell Mol Physiol.

[CR44] Berk AJ (2005). Recent lessons in gene expression, cell cycle control, and cell biology from adenovirus. Oncogene.

[CR142] Bernstock JD (2017). Topotecan is a potent inhibitor of SUMOylation in glioblastoma multiforme and alters both cellular replication and metabolic programming. Sci Rep.

[CR17] Bohren KM (2004). A M55V polymorphism in a novel SUMO gene (SUMO-4) differentially activates heat shock transcription factors and is associated with susceptibility to type I diabetes mellitus. J Biol Chem.

[CR34] Buczynski BW, Maduekwe ET, O’Reilly MA (2013). The role of hyperoxia in the pathogenesis of experimental BPD. Semin Perinatol.

[CR20] Cappadocia L, Lima CD (2018). Ubiquitin-like protein conjugation: structures, Chemistry, and mechanism. Chem Rev.

[CR93] Carbia-Nagashima A (2007). RSUME, a small RWD-containing protein, enhances SUMO conjugation and stabilizes HIF-1alpha during hypoxia. Cell.

[CR26] Cassel TN, Nord M (2003). C/EBP transcription factors in the lung epithelium. Am J Physiol Lung Cell Mol Physiol.

[CR28] Cassel TN (2000). C/EBPalpha and C/EBPdelta activate the clara cell secretory protein gene through interaction with two adjacent C/EBP-binding sites. Am J Respir Cell Mol Biol.

[CR15] Celen AB, Sahin U (2020). Sumoylation on its 25th anniversary: mechanisms, pathology, and emerging concepts. FEBS J.

[CR63] Chakraborty K (2017). The mito-DAMP cardiolipin blocks IL-10 production causing persistent inflammation during bacterial pneumonia. Nat Commun.

[CR7] Chang HM, Yeh ETH (2020). SUMO: from bench to Bedside. Physiol Rev.

[CR30] Chen YD (2017). Functional roles of C/EBPalpha and SUMO–modification in lung development. Int J Mol Med.

[CR94] Cheng J (2007). SUMO-specific protease 1 is essential for stabilization of HIF1alpha during hypoxia. Cell.

[CR3] Collaborators GBDCRD (2020). Prevalence and attributable health burden of chronic respiratory diseases, 1990–2017: a systematic analysis for the global burden of Disease Study 2017. Lancet Respir Med.

[CR126] Constanzo JD (2016). PIAS1-FAK Interaction promotes the survival and progression of Non-Small Cell Lung Cancer. Neoplasia.

[CR40] De La Cruz-Herrera CF (2018). A genome-wide screen of Epstein-Barr virus proteins that modulate host SUMOylation identifies a SUMO E3 ligase conserved in herpesviruses. PLoS Pathog.

[CR38] Dong W (2021). Role of the SENP1-SIRT1 pathway in hyperoxia-induced alveolar epithelial cell injury. Free Radic Biol Med.

[CR46] Doucas V (1996). Adenovirus replication is coupled with the dynamic properties of the PML nuclear structure. Genes Dev.

[CR55] Fan Z (2006). SARS-CoV nucleocapsid protein binds to hUbc9, a ubiquitin conjugating enzyme of the sumoylation system. J Med Virol.

[CR48] Fiedler M et al. *Protein-protein interactions facilitate E4orf6-Dependent regulation of E1B-55K SUMOylation in HAdV-C5 infection*. Viruses, 2022. 14(3). 10.3390/v14030463 PMC8953357 35336871

[CR19] Flotho A, Melchior F (2013). Sumoylation: a regulatory protein modification in health and disease. Annu Rev Biochem.

[CR91] Fu D (2008). Expression and role of factor inhibiting hypoxia-inducible factor-1 in pulmonary arteries of rat with hypoxia-induced hypertension. Acta Biochim Biophys Sin (Shanghai).

[CR97] G SC et al. *Arginase as a potential biomarker of Disease Progression: a Molecular Imaging Perspective*. Int J Mol Sci, 2020. 21(15). 10.3390/ijms21155291 PMC7432485 32722521

[CR136] Gao C (2022). SENP1 inhibition suppresses the growth of lung cancer cells through activation of A20-mediated ferroptosis. Ann Transl Med.

[CR13] Gareau JR, Lima CD (2010). The SUMO pathway: emerging mechanisms that shape specificity, conjugation and recognition. Nat Rev Mol Cell Biol.

[CR64] Garg M (2021). Cardiolipin-mediated PPARgamma S112 phosphorylation impairs IL-10 production and inflammation resolution during bacterial pneumonia. Cell Rep.

[CR106] Ghatak S (2017). Transforming growth factor β1 (TGFβ1)-induced CD44V6-NOX4 signaling in pathogenesis of idiopathic pulmonary fibrosis. J Biol Chem.

[CR84] Gross TJ (2014). A microRNA processing defect in smokers’ macrophages is linked to SUMOylation of the endonuclease DICER. J Biol Chem.

[CR9] Guo D (2004). A functional variant of SUMO4, a new I kappa B alpha modifier, is associated with type 1 diabetes. Nat Genet.

[CR96] Hara M (2020). Arginase 2 is a mediator of ischemia-reperfusion injury in the kidney through regulation of nitrosative stress. Kidney Int.

[CR72] Harada M (2009). Functional analysis of the thymic stromal lymphopoietin variants in human bronchial epithelial cells. Am J Respir Cell Mol Biol.

[CR21] Herriges M, Morrisey EE (2014). Lung development: orchestrating the generation and regeneration of a complex organ. Development.

[CR42] Hofmann S (2021). Double-edged role of PML nuclear bodies during human adenovirus infection. Virus Res.

[CR118] Hou G (2021). SUMOylation of YTHDF2 promotes mRNA degradation and cancer progression by increasing its binding affinity with m6A-modified mRNAs. Nucleic Acids Res.

[CR127] Hung PF (2019). Hypoxia-induced slug SUMOylation enhances lung cancer metastasis. J Exp Clin Cancer Res.

[CR107] Imoto S (2008). Sumoylation of Smad3 stimulates its nuclear export during PIASy-mediated suppression of TGF-beta signaling. Biochem Biophys Res Commun.

[CR105] Inui N, Sakai S, Kitagawa M. *Molecular Pathogenesis of Pulmonary Fibrosis, with Focus on Pathways related to TGF-β and the ubiquitin-proteasome pathway*. Int J Mol Sci, 2021. 22(11). 10.3390/ijms22116107 PMC8201174 34198949

[CR87] Jiang Y (2015). Increased SUMO-1 expression in response to hypoxia: Interaction with HIF-1alpha in hypoxic pulmonary hypertension. Int J Mol Med.

[CR86] Jin H (2014). Melatonin attenuates hypoxic pulmonary hypertension by inhibiting the inflammation and the proliferation of pulmonary arterial smooth muscle cells. J Pineal Res.

[CR60] Jin S (2022). Suppression of ACE2 SUMOylation protects against SARS-CoV-2 infection through TOLLIP-mediated selective autophagy. Nat Commun.

[CR74] Jones B (2017). Animal models of COPD: what do they tell us?. Respirology.

[CR39] K ST (2021). SUMO and SUMOylation pathway at the forefront of host Immune Response. Front Cell Dev Biol.

[CR111] Ke C (2019). SUMO1 promotes the proliferation and invasion of non-small cell lung cancer cells by regulating NF-κB. Thorac Cancer.

[CR145] Kho C (2015). Small-molecule activation of SERCA2a SUMOylation for the treatment of heart failure. Nat Commun.

[CR54] Kim JM (2020). Identification of Coronavirus isolated from a patient in Korea with COVID-19. Osong Public Health Res Perspect.

[CR141] Kim HS, et al. TAK-981, a SUMOylation inhibitor, suppresses AML growth immune-independently. Blood Adv; 2023. 10.1182/bloodadvances.2022007956 PMC10338213 36809797

[CR130] Kluge A (2011). Protein inhibitor of activated STAT3 expression in lung cancer. Mol Oncol.

[CR140] Langston SP (2021). Discovery of TAK-981, a first-in-class inhibitor of SUMO-Activating enzyme for the treatment of Cancer. J Med Chem.

[CR109] Lear T, Chen BB (2016). Therapeutic targets in fibrotic pathways. Cytokine.

[CR108] Lear T (2016). Ubiquitin E3 ligase FIEL1 regulates fibrotic lung injury through SUMO-E3 ligase PIAS4. J Exp Med.

[CR4] Lee JM (2023). Control of protein stability by post-translational modifications. Nat Commun.

[CR10] Lhatoo S (2015). Sudden unexpected death in epilepsy: identifying risk and preventing mortality. Epilepsia.

[CR52] Li SJ, Hochstrasser M (1999). A new protease required for cell-cycle progression in yeast. Nature.

[CR56] Li FQ (2005). Sumoylation of the nucleocapsid protein of severe acute respiratory syndrome coronavirus. FEBS Lett.

[CR121] Li H (2013). Ubc9 promotes invasion and metastasis of lung cancer cells. Oncol Rep.

[CR114] Li Y (2021). LncRNA SNHG3 promotes proliferation and metastasis of Non-Small-Cell Lung Cancer cells through miR-515-5p/SUMO2 Axis. Technol Cancer Res Treat.

[CR16] Liang YC (2016). SUMO5, a Novel Poly-SUMO isoform, regulates PML Nuclear Bodies. Sci Rep.

[CR71] Liang S (2022). CBX4 regulates long-form thymic stromal lymphopoietin-mediated airway inflammation through SUMOylation in House Dust Mite-induced asthma. Am J Respir Cell Mol Biol.

[CR31] Lignelli E (2019). Recent advances in our understanding of the mechanisms of lung alveolarization and bronchopulmonary dysplasia. Am J Physiol Lung Cell Mol Physiol.

[CR119] Liu X (2015). Knockdown of SUMO-activating enzyme subunit 2 (SAE2) suppresses cancer malignancy and enhances chemotherapy sensitivity in small cell lung cancer. J Hematol Oncol.

[CR100] Liu Y (2017). Manipulating PML SUMOylation via silencing UBC9 and RNF4 regulates Cardiac Fibrosis. Mol Ther.

[CR134] Liu K, Zhang J, Wang H (2018). Small ubiquitin-like modifier/sentrin-specific peptidase 1 associates with chemotherapy and is a risk factor for poor prognosis of non-small cell lung cancer. J Clin Lab Anal.

[CR75] Lopez-Campos JL, Tan W, Soriano JB (2016). Global burden of COPD. Respirology.

[CR89] Lu Q (2016). Angiogenic factor AGGF1 activates autophagy with an essential role in therapeutic angiogenesis for Heart Disease. PLoS Biol.

[CR29] Luc PV (1996). Transcriptional regulation of the CYP2B1 and CYP2B2 genes by C/EBP-related proteins. Biochem Pharmacol.

[CR82] Lugg ST (2022). Cigarette smoke exposure and alveolar macrophages: mechanisms for lung disease. Thorax.

[CR58] Marra MA (2003). The genome sequence of the SARS-associated coronavirus. Science.

[CR73] Matera MG (2020). TSLP inhibitors for Asthma: current status and future prospects. Drugs.

[CR120] McDoniels-Silvers AL (2002). Differential gene expression in human lung adenocarcinomas and squamous cell carcinomas. Clin Cancer Res.

[CR11] Mendler L, Braun T, Muller S (2016). The Ubiquitin-Like SUMO system and heart function: from development to Disease. Circ Res.

[CR70] Mims JW (2015). Asthma: definitions and pathophysiology. Int Forum Allergy Rhinol.

[CR61] Mizgerd JP (2008). Acute lower respiratory tract infection. N Engl J Med.

[CR143] Mo YY (2002). Nucleolar delocalization of human topoisomerase I in response to topotecan correlates with sumoylation of the protein. J Biol Chem.

[CR32] Morty RE (2018). Recent advances in the pathogenesis of BPD. Semin Perinatol.

[CR123] Moschos SJ (2010). Expression analysis of Ubc9, the single small ubiquitin-like modifier (SUMO) E2 conjugating enzyme, in normal and malignant tissues. Hum Pathol.

[CR133] Mu J (2014). Over-expression of small ubiquitin-like modifier proteases 1 predicts chemo-sensitivity and poor survival in non-small cell lung cancer. Chin Med J (Engl).

[CR49] Muncheberg S et al. *E1B-55K-Mediated regulation of RNF4 SUMO-Targeted ubiquitin ligase promotes human adenovirus gene expression*. J Virol, 2018. 92(13). 10.1128/JVI.00164-18 PMC6002701 29695423

[CR95] Murata T (2002). Decreased endothelial nitric-oxide synthase (eNOS) activity resulting from abnormal interaction between eNOS and its regulatory proteins in hypoxia-induced pulmonary hypertension. J Biol Chem.

[CR12] Namuduri AV (2017). A Proteomic Approach to identify alterations in the small ubiquitin-like modifier (SUMO) network during controlled mechanical ventilation in Rat Diaphragm muscle. Mol Cell Proteomics.

[CR18] Nayak A, Müller S (2014). SUMO-specific proteases/isopeptidases: SENPs and beyond. Genome Biol.

[CR66] Nie X (2016). Aspergillus flavus SUMO contributes to fungal virulence and toxin attributes. J Agric Food Chem.

[CR144] Niu Q (2022). Antileukemic effects of topoisomerase I inhibitors mediated by de-SUMOylase SENP1. Biochim Biophys Acta Mol Basis Dis.

[CR33] Obst S et al. *Perinatal Hyperoxia and Developmental Consequences on the Lung-Brain Axis* Oxid Med Cell Longev, 2022. 2022: p. 5784146. 10.1155/2022/5784146 PMC8894035 35251477

[CR131] Ogata Y (2006). Overexpression of PIAS3 suppresses cell growth and restores the drug sensitivity of human lung cancer cells in association with PI3-K/Akt inactivation. Neoplasia.

[CR6] Okura T (1996). Protection against Fas/APO-1- and tumor necrosis factor-mediated cell death by a novel protein, sentrin. J Immunol.

[CR98] Pandey D (2018). Hypoxia triggers SENP1 (sentrin-Specific protease 1) modulation of KLF15 (Kruppel-Like factor 15) and transcriptional regulation of Arg2 (arginase 2) in Pulmonary Endothelium. Arterioscler Thromb Vasc Biol.

[CR83] Park JE (2011). Dicer recognizes the 5’ end of RNA for efficient and accurate processing. Nature.

[CR62] Pascual G (2005). A SUMOylation-dependent pathway mediates transrepression of inflammatory response genes by PPAR-gamma. Nature.

[CR50] Pennella MA (2010). Adenovirus E1B 55-kilodalton protein is a p53-SUMO1 E3 ligase that represses p53 and stimulates its nuclear export through interactions with promyelocytic leukemia nuclear bodies. J Virol.

[CR69] Pereira RV (2011). Molecular characterization of SUMO E2 conjugation enzyme: differential expression profile in Schistosoma mansoni. Parasitol Res.

[CR68] Pereira RV et al. *Transcriptional Profile and Structural Conservation of SUMO-Specific Proteases in Schistosoma mansoni* J Parasitol Res, 2012. 2012: p. 480824. 10.1155/2012/480824 PMC3483780 23125916

[CR67] Pereira RV (2014). Up-regulation of SUMO E3 ligases during lung schistosomula and adult worm stages. Parasitol Res.

[CR25] Ramji DP, Foka P (2002). CCAAT/enhancer-binding proteins: structure, function and regulation. Biochem J.

[CR43] Regad T, Chelbi-Alix MK (2001). Role and fate of PML nuclear bodies in response to interferon and viral infections. Oncogene.

[CR41] Ribet D, Cossart P (2018). Ubiquitin, SUMO, and NEDD8: key targets of bacterial pathogens. Trends Cell Biol.

[CR99] Richeldi L, Collard HR, Jones MG (2017). Idiopathic Pulmonary Fibrosis Lancet.

[CR65] Sa-Pessoa J et al. *Klebsiella pneumoniae reduces SUMOylation to limit host defense responses*. mBio, 2020. 11(5). 10.1128/mBio.01733-20 PMC7527722 32994335

[CR14] Saitoh H, Hinchey J (2000). Functional heterogeneity of small ubiquitin-related protein modifiers SUMO-1 versus SUMO-2/3. J Biol Chem.

[CR1] Schluger NW, Koppaka R (2014). Lung disease in a global context. A call for public health action. Ann Am Thorac Soc.

[CR59] Shang J (2020). Cell entry mechanisms of SARS-CoV-2. Proc Natl Acad Sci U S A.

[CR22] Shannon JM, Hyatt BA (2004). Epithelial-mesenchymal interactions in the developing lung. Annu Rev Physiol.

[CR2] Sharma A, Ahmad Farouk I, Lal SK. *COVID-19: a review on the Novel Coronavirus Disease Evolution, Transmission, Detection, Control and Prevention*. Viruses, 2021. 13(2). 10.3390/v13020202 PMC7911532 33572857

[CR81] Shaykhiev R (2009). Smoking-dependent reprogramming of alveolar macrophage polarization: implication for pathogenesis of chronic obstructive pulmonary disease. J Immunol.

[CR5] Song L, Luo ZQ (2019). Post-translational regulation of ubiquitin signaling. J Cell Biol.

[CR78] Song Y (2015). Carbocysteine restores steroid sensitivity by targeting histone deacetylase 2 in a thiol/GSH-dependent manner. Pharmacol Res.

[CR79] Song Y (2019). Carbocisteine improves histone deacetylase 2 Deacetylation Activity via regulating sumoylation of histone deacetylase 2 in human tracheobronchial epithelial cells. Front Pharmacol.

[CR47] Stubbe M et al. *Viral DNA binding protein SUMOylation promotes PML nuclear body localization next to viral replication Centers*. mBio, 2020. 11(2). 10.1128/mBio.00049-20 PMC7078464 32184235

[CR113] Su X (2019). Expression of SUMO1P3 compared with SUMO1 is an independent predictor of patient outcome in Lung Adenocarcinoma. Med Sci Monit.

[CR125] Sulzmaier FJ, Jean C, Schlaepfer DD (2014). FAK in cancer: mechanistic findings and clinical applications. Nat Rev Cancer.

[CR128] Sun L (2013). A SUMOylation-dependent pathway regulates SIRT1 transcription and lung cancer metastasis. J Natl Cancer Inst.

[CR103] Sun W (2022). SENP1 regulates the transformation of lung resident mesenchymal stem cells and is associated with idiopathic pulmonary fibrosis progression. Cell Commun Signal.

[CR37] Tan F (2018). Attenuated SUMOylation of sirtuin 1 in premature neonates with bronchopulmonary dysplasia. Mol Med Rep.

[CR132] Tantai J, Pan X, Hu D (2016). RNF4-mediated SUMOylation is essential for NDRG2 suppression of lung adenocarcinoma. Oncotarget.

[CR23] Wan XQ (2019). SENP1 has an important role in lung development and influences the differentiation of alveolar type 2 cells. Int J Mol Med.

[CR124] Wang ZG (1998). Role of PML in cell growth and the retinoic acid pathway. Science.

[CR137] Wang RT (2013). Inhibition of SENP1 induces radiosensitization in lung cancer cells. Exp Ther Med.

[CR138] Wang J (2013). Integrative genomics analysis identifies candidate drivers at 3q26-29 amplicon in squamous cell carcinoma of the lung. Clin Cancer Res.

[CR102] Wang X (2018). Expression, purification, and evaluation of in vivo anti-fibrotic activity for soluble truncated TGF-β receptor II as a cleavable His-SUMO fusion protein. World J Microbiol Biotechnol.

[CR51] Wimmer P, Schreiner S (2015). Viral mimicry to usurp ubiquitin and SUMO host pathways. Viruses.

[CR85] Wright JL, Levy RD, Churg A (2005). Pulmonary hypertension in chronic obstructive pulmonary disease: current theories of pathogenesis and their implications for treatment. Thorax.

[CR122] Wu F (2009). MicroRNA-mediated regulation of Ubc9 expression in cancer cells. Clin Cancer Res.

[CR36] Yang K, Dong W (2021). SIRT1-Related signaling pathways and their Association with Bronchopulmonary Dysplasia. Front Med (Lausanne).

[CR110] Yang S (2021). Therapeutic applications of mesenchymal stem cells in idiopathic pulmonary fibrosis. Front Cell Dev Biol.

[CR135] Yang Q (2021). SENP1 Aberrance and its linkage to clinical features, adjuvant regimen, and prognosis in patients with Surgical Non-small Cell Lung Cancer receiving adjuvant chemotherapy. Front Surg.

[CR88] Yao Y (2019). SUMOylation of Vps34 by SUMO1 promotes phenotypic switching of vascular smooth muscle cells by activating autophagy in pulmonary arterial hypertension. Pulm Pharmacol Ther.

[CR45] Yousef AF (2010). Identification of a molecular recognition feature in the E1A oncoprotein that binds the SUMO conjugase UBC9 and likely interferes with polySUMOylation. Oncogene.

[CR104] Yu L et al. *Ginkgolic acid improves bleomycin-induced pulmonary fibrosis by inhibiting SMAD4 SUMOylation* Oxid Med Cell Longev, 2022. 2022: p. 8002566. 10.1155/2022/8002566 PMC9192210 35707278

[CR115] Zahra K (2020). Pyruvate kinase M2 and Cancer: the role of PKM2 in promoting Tumorigenesis. Front Oncol.

[CR112] Zhang Y (2019). SUMO1P3 is associated clinical progression and facilitates cell migration and invasion through regulating miR-136 in non-small cell lung cancer. Biomed Pharmacother.

[CR101] Zhao W (2021). Upregulation of small Ubiquitin-Like modifier 2 and protein SUMOylation as a cardioprotective mechanism against myocardial ischemia-reperfusion Injury. Front Pharmacol.

[CR90] Zhou F (2016). SENP–1 enhances hypoxia–induced proliferation of rat pulmonary artery smooth muscle cells by regulating hypoxia–inducible factor–1alpha. Mol Med Rep.

[CR57] Zhou P (2020). A pneumonia outbreak associated with a new coronavirus of probable bat origin. Nature.

[CR76] Zhou H (2020). Cigarette smoke extract stimulates bronchial epithelial cells to undergo a SUMOylation turnover. BMC Pulm Med.

[CR117] Zhu T (2014). Crystal structure of the YTH domain of YTHDF2 reveals mechanism for recognition of N6-methyladenosine. Cell Res.

[CR35] Zhu Y (2020). Sumoylation of CCAAT-enhancer-binding protein alpha inhibits lung differentiation in Bronchopulmonary Dysplasia model rats. J Cell Mol Med.

[CR53] Ziegler CGK (2020). SARS-CoV-2 receptor ACE2 is an Interferon-Stimulated gene in human airway epithelial cells and is detected in specific cell subsets across tissues. Cell.

